# Stainless steel versus titanium volar multi-axial locking plates for fixation of distal radius fractures: a randomised clinical trial

**DOI:** 10.1186/1471-2474-15-74

**Published:** 2014-03-11

**Authors:** Gregory B Couzens, Susan E Peters, Kenneth Cutbush, Benjamin Hope, Fraser Taylor, Christopher D James, Carly R Rankin, Mark Ross

**Affiliations:** 1 Brisbane Hand and Upper Limb Research Institute, 9/259 Wickham Tce, Brisbane, QLD 4001, Australia; 2 Orthopaedic Department, Princess Alexandra Hospital, 199 Ipswich Road, Woolloongabba, QLD 4102, Australia; 3 University of Queensland, St Lucia, QLD 4067, Australia

**Keywords:** Distal radius, Randomized trial, Volar plating, Trimed, Medartis, Wrist, Surgery

## Abstract

**Background:**

Distal radius fractures are among the most common fractures seen in the hospital emergency department. Of these, over 40% are considered unstable and require some form of fixation. In recent years with the advent of low profile plating, open reduction and internal fixation (ORIF) using volar plates has become the surgical treatment of choice in many hospitals. However, it is currently unknown which plating system has the lowest complication rate and/or superior clinical and radiological outcomes following surgery. Few studies have compared different types of plates, which may have various features, different plate and screw designs or may be manufactured from different materials (for example, stainless steel or titanium). This study will specifically investigate and compare the clinical and radiological outcomes and complication rates of two commonly used volar plating systems for fixation of distal radius fractures: one made from stainless steel (Trimed™ Volar Plate, Trimed™, California, USA) and the other made from titanium (Medartis® Aptus Volar Plate, Medartis®, Basel, Switzerland). The primary aim of this study is to determine if there is a difference on the Patient Reported Wrist Evaluation six months following ORIF using a volar plate for adult patients with a distal radius fracture.

**Methods/Design:**

This study will implement a randomized prospective clinical trial study design evaluating the outcomes of two different types of volar plates: one plate manufactured from stainless steel (Trimed™ Volar Plate) and one plate manufactured from titanium (Medartis® Aptus Volar Plate). The surgery will be performed at a major trauma hospital in Brisbane, Australia. Outcome measures including function, adverse events, range of movement, strength, disability, radiological findings and health-related quality of life will be collected at 6 weeks, 3, 6, 12 and 24 months following surgery. A parallel economic analysis will also be performed. This randomized clinical trial is due to deliver results in December 2016.

**Discussion:**

Results from this trial will contribute to the evidence on operative management of distal radius fractures and plate material type.

**Trial registration:**

ACTRN12612000969864

## Background

Distal radius fractures are the most common type of all fractures seen in the emergency department and constitute 10% to 25% of all extremity fractures [1,2]. The incidence of distal radius fractures continues to rise as the population in industrialised countries grows in line with an increase in age and life expectancies [3,4]. The occurrence of distal radius fractures peaks within two different age groups: high energy trauma, typically in males aged 5–24 years, or low-energy injuries commonly seen in the elderly female population aged 65 years and older [5].

The treatment of distal radius fractures has developed greatly over recent years with a move towards treating these fractures by means of internal fixation. Although closed reduction and casting remain viable options for some non-displaced simple fractures of the distal radius, the role of ORIF continues to grow due to its ability to more reliably restore wrist anatomy, minimize immobilization and establish acceptable clinical outcomes [4]. Numerous methods of surgical stabilisation exist including: manipulation with Kirschner wires (k-wires), external fixators, dorsal and volar plating and fragment specific fixation. All have been found to be successful in maintaining correction of the reduced fracture however volar plating has more recently become the favourable choice for managing distal radius fractures especially in more complex fracture patterns and/or osteoporotic bone [6-8]. The advent of variable angle volar plating systems has also allowed cases with increasing comminution and displacement, poor bone quality, and dorsally angulated fractures, to be fixed with less tendon irritation and the reduced need for hardware removal [8,9]. In addition, volar plating allows restoration of anatomy, stable fixation, reduced periods of immobilisation and earlier return to function [6,8]. As the popularity of volar plating has increased so has the number and type of volar plating systems available to orthopaedic surgeons. Differences range from the plate design (i.e. shape and contour), material, type, locking screw mechanism and number of screws used. The general benefits of variable angle plating systems have been reported to include [8]:

•Flexible deployment with respect to variations in radial size;

•Accommodation of proximal/distal variation in volar fracture lines;

•Accommodation of medial and lateral variation in fracture lines;

•Adaptation of screw direction to specific fracture fragments.

A recent literature review conducted by Gehrmann et al. on distal radius fracture management in the elderly, found that patients with higher demands benefit from fracture stabilisation using locking volar plates [3]. Volar plating with fixed-angle screws may also be particularly suitable for elderly patients whose fractures take longer to heal or are more susceptible to pin-site infection.

Additionally, newer features of volar plates, such as locking and variable angle mechanisms, have been thought to further reduce complication rates and improve effectiveness in patients of all ages [6]. This remains controversial however, as a number of complications including loss of fixation, tendon irritation or rupture, median nerve complications and distal radial ulnar joint dysfunction, have been reported in the literature [10,11]. Various factors, such as plate and screw type, have been found to contribute to this complication rate, which highlights the importance of investigation into clinical and radiological outcomes of surgery for this patient population.

Comparison of complications between dorsal and volar plates has been examined previously with results indicating a higher rate of association between volar plates and neuropathic complications but less tendon irritation or ruptures than dorsal plates [2]. A study by Soong et al. also found that very few complications were recorded in their cohort of distal radius fractures fixed with volar plates, compared with dorsal plates [11].

A retrospective study of 115 patients with comminuted intra-articular distal radius fractures was performed by Richards et al. who compared radiographic and clinical outcomes of patients treated with external fixation to those treated with volar plate internal fixation [10]. They reported fewer complications, better range of movement, pain and functional scores in the ORIF group.

A randomised clinical trial of 53 patients comparing external fixation and ORIF (either dorsal or volar plates) by Grewal and colleagues found that ORIF had significantly lower Patient Rated Wrist Evaluation (PRWE) scores across all time points (3, 6, 12 months) with better outcomes observed in the volar plating group [12].

Park et al. investigated the clinical outcomes of a consecutive cohort of 20 patients undergoing ORIF of their distal radius fractures using the Medartis Aptus Volar Plate up to a one year period [4]. They found that all fractures healed in every case by 12 weeks post-operation with no loss of reduction in 19 cases (of 20 patients). Range of movement, grip strength and Disability of the Arm, Shoulder and Hand (DASH) scores were all within acceptable ranges.

Previous studies have investigated the clinical and radiological outcomes of volar plating systems in cohort series designs [9,13,14]. Osada et al. found in their series of 49 fractures in 49 patients fixed with volar plates, good radiological results, good to excellent Gartland and Werley scores, low DASH scores, a high degree of patient satisfaction and no record of complications except plate removal [13]. A total of three plates were removed between six and 10 months postoperatively. Two plates were removed due to patient request and the other was removed because the distal locking pins appeared to be intra-articular on the computed tomography scan [9]. Sanchez-Crespo retrospectively reviewed 145 patients (fixed with either a Medartis Aptus Volar 2.5 mm Plate or Synthes 2.4mmLCP distal radius plate) and analysed results of 95 of these patients [14]. They found good mean functional scores using the PRWE (mean 13; range 0–64) however 8% of patients presented with a complication (e.g. chronic pain, malunion, tendon tears, carpal tunnel syndrome, or requiring hardware removal).

Titanium and stainless steel volar plating systems for distal radius fractures are both readily available and in common use in orthopaedic surgery. Titanium implants have been reported to have benefits including reduced implant stiffness, increased biocompatibility and diminished stress shielding [15]. However tenosynovitis and extensor tendon ruptures have been reported in the use of low profile titanium plates used for dorsal fixation of distal radius fractures [16]. Conversely, stainless steel plates have been thought to have less tendon irritation and adhesions when used in wrist surgery [16].

Recently, Souer et al. specifically investigated the clinical and radiological outcomes comparing 2.4 mm titanium and 3.5 mm stainless steel volar plates and found improved range of movement in the group that received the 2.4 mm titanium plate at 12 and 24 months post-surgery [13]. However, patients in the 3.5 mm stainless steel volar plate group had better radiological outcomes at all time points. Along with the obvious limitations in the study design (retrospective review of a prospective cohort), sample size (n = 62), and high loss to follow-up, the results could be attributed to either the plate thickness or material type. To date, no randomized trials are known to exist comparing material type (i.e. stainless steel versus titianium) of volar plates.

The research question for this study is: Do patients who have their distal radius fractures internally fixated with a titanium plate have the same outcomes as patients who have their distal radius fractures internally fixated with a stainless steel plate?

## Methods/Design

### Design

This is a prospective double blind randomized clinical trial. This study was approved by the Metro South Human Research Ethics Committee (Ref: HREC/12/QPAH/293) and registered with the Australian New Zealand Clinical Trials Registry (ANZCTR) (Ref: ACTRN12612000969864).

### Study participants

Participants will be selected for inclusion in the study if:

1) Aged 18 years and over; male or female;

2) Acute (defined as surgery needs to take place within 3 weeks of injury) distal radius fracture as diagnosed on x-ray and/or CT scan with surgical fixation.

Participants will be excluded from the study if they present with a:

1) History of previous wrist fracture, injury or surgery in the same wrist with ongoing symptoms or functional limitation at the time of the distal radius fracture;

2) Significant acute associated trauma or injuries to the ipsilateral upper limb;

3) Associated significant other injuries increasing risk of surgery or preventing compliance with rehabilitation protocol (e.g. traumatic brain injury; spinal cord injury; traumatic upper extremity surgery);

4) Wrist fractures that are unable to be fixed adequately with only a volar plate (e.g. requires additional fixation). If this is determined intra-operatively, patients will not be progressed further into the study;

5) Medical/Anaesthetic contraindications to surgery;

6) Unable to comply with rehabilitation or attend follow up appointments as determined at the time of surgery (e.g. resides internationally);

7) Are currently pregnant.

### Trial interventions

The interventions being compared are:

Intervention A: Early surgical intervention using a Stainless Steel volar locking plate.

Intervention B: Early surgical intervention using a Titanium volar locking plate.

The hospital involved in the trial currently use both types of plate for open reduction and internal fixation of distal radius fractures and all of the surgeons involved are surgically experienced with volar plate fixation for distal radius fractures. The operative technique takes place under general anesthetic and each patient will undergo the surgery according to their random allocation of Intervention A or Intervention B. Randomisation will occur at the time of surgery booking after the patients has been appropriately consented and enrolled into the study.

All patients undergo general anaesthetic and high arm tourniquet control. The approach is via a volar incision and then through the bed of the flexor carpi radialis tendon. The plane between the flexor tendons and pronator quadratus is identified and pronator quadratus is then dissected carefully as a flap from the radial side to expose the underlying distal radius. Brachioradialis is released completely due to its adverse coronal plane deforming force upon the fracture.

Reduction of the fracture is dependent upon the fracture pattern and may be indirect with traction and manipulation, or direct with mobilization of the fragments directly with k-wires or bone punch. After the fracture has been initially temporarily stabilized with k-wires the volar multi-axial locking plate is applied and secured with screws to gain control of the radial and ulnar columns. It is paramount to ensure that the ulnar column is held securely to prevent any ulnar translocation. In the majority of cases the technique during surgery is to insert the variable angle distal row screws first according to the surgeons technique and to place them in the most appropriate subchondral configuration at the most appropriate depth whilst accommodating the variable fracture fragment configuration. The plate is reduced to the shaft and the diaphyseal screws are then inserted whilst fine-tuning fracture reduction [8]. If there is any significant bone loss the void is filled using calcium phosphate injectable bone substitute.

Most fractures are amenable to this single plate fixation, but some complex fractures require specific fragment plating techniques with additional metalwork insertion and if this is required intra-operatively then the patient will be excluded and will not continue further in the study. Occasionally, patients require a small dorsal incision and arthrotomy to aid the fracture reduction and as long as this is only for reduction purposes they may remain in the study.

Closure of the wound begins initially with suturing of pronator quadratus back to its original position where possible. In the majority of cases, even when the pronator quadratus is lacerated by the fracture there will be sufficient tissue available for repair to cover the most distal aspect of the plate. This soft tissue layer can prevent flexor tendon irritation on the distal plate. Subcuticular and skin suturing technique is down to the surgeon’s preference. The wound is dressed with a waterproof dressing and then a bulky dressing in neutral position is applied to the wrist, allowing full range of movement of the fingers including metacarpal phalangeal joint flexion. The patient is given a sling for comfort and to elevate the hand [8].

#### Rehabilitation

Hand Therapy intervention will be performed by Occupational Therapists who are experienced in treating distal radius fractures in hospital outpatient departments. Therapy will commence within 3 days of the operation. At this time the patients will have a thermoplastic static volar wrist orthosis fabricated and commence an early active mobilisation program as detailed in Table 1. Any deviation from the study protocol will be documented to control for in the analysis.

**Table 1:** Rehabilitation protocol post-operation

**Time frame**	**Treatment guideline**
Day 1–2 post-op	**Advice and education**
	• Education regarding injury/fixation/and rehabilitation protocol- minimal axial loading
	• Non-loaded ADL’s with splint off
	**Wound**
	**•** Remove post-operative dressing. Redress with simple dressing
	**Oedema**
	• Elevation and retrograde massage for oedema.
	• Application of compression to manage oedema as required
	**Orthosis**
	• Fabrication of thermoplastic static volar wrist orthosis in extension ( ≥15 degrees), ensuring full digit flexion achievable
	**Exercises**
	Commence active wrist ROM: dart throwing axis; wrist flexion/extension/pronation/supination/radial & ulnar deviation; finger tendon gliding; thumb extension, opposition
	• Commence active assist wrist extension/supination
	• AROM non-affected joints of upper extremity
Day 10	• Removal of sutures
	• Continue wrist orthosis
	• Commence scar management: massage, desensitization and application of silicone based products
	• Oedema Management as required: retrograde massage, compression, elevation
	• Continue exercise program
Weeks 2–3	• Continue wrist orthosis
	• Continue scar management
	• Ongoing oedema management as required
	• Light function in splint
	• Active assisted and passive wrist exercises
	• If significant difficulty achieving supination apply gentle tension rotational splint for Pronation/Supination. Commence dynamic rotational orthosis if required dependent on fracture healing, pain and oedema
	• Commence stretch with 500 g weight and heat.
Weeks 4–5	• Continue wrist orthosis for at risk activities only
	• Continue oedema and scar management as required.
	• Active, active-assisted, passive wrist extension/flexion/pronation/supination
	• Continue weighted stretches and dynamic orthotic use as required
Week 6	• Cease wrist orthosis
	• Commence grip and wrist strengthening (dependant on fracture healing)
	• Increase functional activity with affected hand
	• Dynamic orthotic to increase range of movement
	• Gradual increase in strengthening program
	• Wrist proprioception exercises
Week 8 weeks +	• Continue dynamic orthotic use as required
	• Continue active and passive wrist exercises
	• Ongoing strengthening program
	• Gradual increase with weight bearing, heavy lifting
	• Function/work hardening

#### Radiological assessment

Normal radiological assessment of the wrist using either x-ray or computed tomography scans will be performed. Diagnosis will be confirmed with x-ray or computed tomography scan. X-rays will be routinely taken post-operatively at 2 weeks, 6 weeks and thereafter to assess bony fracture union and any fracture or hardware related complications as required. An X-ray will also be taken at final follow-up at two years to assess for any bony or hardware related complications. Radiographic assessments will not be blinded.

#### Outcome measures

A suite of outcome measures recommended and previously used in research for this patient group will be used [7,9,13]. A summary of these outcome measures and the tool used are detailed in Table 2.

**Table 2:** Outcome measurement tools

**Outcome**	**Assessment**
Function	Patient rated wrist evaluation*
Disability	Disabilities of the arm, shoulder and hand
Health-related quality of life	EQ-5D-3 L (Australian)
Pain	Visual analogue scale
Satisfaction	Visual analogue scale
Grip strength	Jamar dynamometer
Range of movement	Goniometric evaluation
Radiological outcomes	Study specific generated checklist
Adverse events	Study specific generated checklist

*Primary outcome measure.

Patient demographic information and baseline (pre-injury) functional status will be collected after consent to take part in the trial. Structured information regarding other injuries which may affect outcome e.g. disruption of the carpal ligaments, will be collected but all patients will be included in the analysis.

The primary outcome measure for this study is the:

### Patient rated wrist evaluation (PRWE)

The PRWE is a 15-item questionnaire designed to measure wrist pain and disability in activities of daily living. The PRWE allows patients to rate their levels of wrist pain and disability from 0 to 10, (0 = no difficulty, 10 = unable to do) and consists of 2 subscales:

1) PAIN subscale – 5 items.

2) FUNCTION subscale - 10 items (specific and usual activities).

In addition to the individual subscale scores, a total score can be computed on a scale of 100 (0 = no disability), where pain and function problems are weighted equally.

The PRWE is a standardized outcome tool that is easy to administer and score, and complements traditional impairment and radiographic measures. The PRWE has been used to assess wrist-related pain and disability in various populations and its reliability, validity, and responsiveness have been tested and reported in published studies [17].

The secondary outcome measures in this trial are the:

### Adverse events

The following adverse events will be recorded: significant reduction in movement (6 weeks or greater), high levels of reported pain (2 weeks or greater), significant reduction in function (6 weeks or greater), non-union, malunion, infection, chronic regional pain syndrome, implant failure (eg implant breakage, screw migration, loosening), fracture fixation failure (implant failing to maintain fracture fixation), tendon irritation (3 months or greater), tendon rupture, nerve injury, excessive scarring or hypersensitivity (6 weeks or greater).

### EQ-5D (Euroqol-5D)

The EQ-5D-3 L is a validated, generalized, quality of life questionnaire consisting of 5 domains related to daily activities with a three-level answer possibility. The EQ-5D-3 L obtains information pertaining to the current health status of the participant and measures physical, emotional and social status. The combination of answers leads to a total quality of life score.

### Short form disabilities of arm, shoulder and hand score (QuickDASH)

The QuickDASH is a shortened version of the DASH Outcome Measure. The QuickDASH uses 11 items to measure physical function and symptoms in people with musculoskeletal disorders of the upper limb. The QuickDASH also has two optional modules intended to measure symptoms and function in athletes, performing artists and other workers whose jobs require a high degree of physical performance. These optional models are scored separately. Both the QuickDASH and the full DASH Outcome measure are valid, reliable and responsive and can be used for clinical and/or research purposes [18].

### VAS

The Visual Analogue Scale assesses wrist pain and satisfaction in relation to the distal radius fixation. Participants are requested to mark their response on the 10 cm line (0–100) with a single slash (/). VAS Scales, especially for pain, are widely used and reported.

### Grip strength

Grip strength will be assessed using a Jamar Dynamometer. Three measurements for both the affected and non-affected arms will be recorded and an average of the three measurements will be used in the data analysis.

### Range of movement

Standard goniometric assessment of passive and active wrist movement will be completed according to the American Clinical Guidelines for Assessment of wrist movement. A standard medium sized wrist goniometer will be used.

### Radiographic evaluation

Standard posterior-anterior, lateral and oblique radiograph projection views will be taken to confirm diagnosis pre-operatively. X-rays will then be routinely performed as per the standard level of care to assess for union, standard radiographic parameters and hardware complications. An additional x-ray will also be taken at 24 months post-operation to assess for malunion, radiographic parameters and hardware complications.

These outcomes will be administered by a blinded assessor at the timeframes detailed in Table 3:

**Table 3:** Intervals for outcome measures

**Assessment**	**Baseline**	**2 weeks**	**6 weeks**	**3 months**	**6 months**	**1 year**	**2 years**
**PRWE**			X	X	X	X	X
**Patient satisfaction**			X	X	X	X	X
**Pain**		X	X	X	X	X	X
**Radiological outcomes**	X	X	X	*	*	*	X
**AROM**		X	X	X	X	X	X
**Complications**		X	X	X	X	X	X
**EQ5D**	X		X	X	X	X	X
**Quick DASH**			X	X	X	X	X
**Grip strength**				X	X	X	X

*Only if required as part of treatment/normal care.

#### Sample size

Recruitment of a total of 130 participants (65 in each group) is anticipated to take approximately 104 weeks (assuming a 75% recruitment rate of eligible participants) based on historical emergency department presentation rate of this patient group. After allowing for a 30% loss to follow up, this investigation has 95% power to detect a between group mean difference in the primary outcome function (PRWE) of 10 (SD-15).

#### Randomisation

Patients providing written informed consent for participation in this trial will be randomly assigned to either early intervention group (titianium or stainless steel volar multi-axial locking plates). The randomisation sequence will be computer generated and concealed in sequentially numbered sealed, opaque envelopes by a person, not otherwise associated with this research, to eliminate any risk of randomisation/recruitment bias. Each envelope will contain a sheet of paper with the words either “TITANIUM” or “STAINLESS STEEL”. This randomisation process will occur at the time of surgery booking.

#### Statistical analysis

Baseline demographic and clinical data will be reported using descriptive statistics and tabulated (e.g. Students *t*-test for continuous variables and Pearson chi-square for categorical variables). Between group differences in baseline data will be examined using unpaired conventional tests of hypothesis (such as unpaired t-tests) depending on the nature of the data. Between group and within group differences in outcome measures over time will be examined using a priori unpaired and paired conventional tests of hypothesis (such as Analysis of Variance with simple effects examined using t-tests) depending on the nature of the data. Bonferroni adjustments for multiple comparisons will be made where appropriate to mitigate risk of type-1 error. The complication rates will be reported in terms of frequency. The frequencies of complications will be compared using statistical analysis such as the Pearson chi-square statistic. For missing data values at different time points, a mixed linear regression model for repeated measures will be performed. Subanalysis for age will be undertaken to account for patients with osteoperotic bone.

## Discussion

This randomized clinical trial is due to deliver results in December 2016.

## Abbreviations

AROM: Active range of motion; ANZCTR: Australian New Zealand clinical trials registry; DASH: Disability of the arm, shoulder and hand; EQ-5D: Euroqol-5D; PRWE: Patient rated wrist evaluation; QuickDASH: Short form disabilities of arm, shoulder and hand score; ROM: Range of motion; SD: Standard deviation; VAS: Visual analogue scale.

## Competing interests

This trial is funded through The Brisbane Hand and Upper Limb Research Institute. The Research Institute receives charitable donations from Medartis, LMT (distributor of Trimed), Depuy and Lima. MR has a professional relationship with Trimed and both MR and GC have a professional relationship with LMT and Medartis. SP and CR are employees of the Brisbane Hand and Upper Limb Research Institute.

## Authors’ contributions

GC developed the protocol and is a principal investigator for the trial. MR developed the protocol and is the chief principal investigator for the study. SP developed the protocol and is the trial coordinator. BH, KC and FT developed the protocol. CR developed the protocol and is a member of the trial management group. All authors read and approved the final manuscript.

## Authors information

GC (MBBS, FRACS, FAOrthA), SP (BOccThy Hons, PhD Candidate), KC (MBBS, FRACS, FAOrthA), BH (MBBS, FRACS, FAOrthA), CR (BOccThy Hons, BBehSciPsych, GradDipPsych), FT (BSc, MbChb, FRACS), CJ (MBBS, MCRS), MR (MBBS, FRACS, FAOrthA).

## Pre-publication history

The pre-publication history for this paper can be accessed here:

http://www.biomedcentral.com/1471-2474/15/74/prepub
